# Allergic disorders and their risk factors in primary Sjögren's syndrome

**DOI:** 10.1016/j.waojou.2023.100745

**Published:** 2023-01-27

**Authors:** Misako Higashida-Konishi, Keisuke Izumi, Tatsuya Shimada, Satoshi Hama, Tatsuhiro Oshige, Hisaji Oshima, Yutaka Okano

**Affiliations:** a Division of Rheumatology, Department of Medicine, National Hospital Organization Tokyo Medical Center, 1528902, Higashigaoka 2-5-1, Meguro-ku, Tokyo, Japan; b Division of Rheumatology, Department of Internal Medicine, Keio University School of Medicine, 1608582, Shinanomachi 35, Shinjyuku-ku, Tokyo, Japan

**Keywords:** Allergic rhinitis, Asthma, Drug allergy, Food allergy, Sjögren's syndrome

## Abstract

**Objective:**

This study aimed to evaluate the prevalence of allergic disorders in patients with primary Sjögren's syndrome (pSS), compare it with that of patients with rheumatoid arthritis (RA), and examine the risk factors in patients with pSS.

**Methods:**

We retrospectively examined the records of patients diagnosed with pSS and RA who regularly visited our department between 2010 and 2020. Allergic disorders included drug allergy, food allergy, allergic contact dermatitis (ACD), allergic rhinitis (AR)/allergic conjunctivitis (AC), and asthma.

**Results:**

Patients with pSS (292 patients) had a higher prevalence of food allergy, drug allergy, and AR/AC than those with RA (413 patients). The multivariate analysis revealed that patients with pSS who had drug allergy had a higher prevalence of food allergy, higher eosinophil levels, and higher positivity rates of anti–SS-related antigen A (SSA) antibodies than those without drug allergy; those with food allergy had a higher rate of ACD than those without food allergy and vice versa; those with AR/AC had a higher rate of ACD and asthma and higher eosinophil levels than those without AR/AC; those with asthma had a higher rate of AR/AC than those without asthma.

**Conclusions:**

Patients with pSS had a higher prevalence of allergic disorders than those with RA. Among patients with pSS, the risk factors for drug allergy were food allergy, higher eosinophil levels, and positivity for anti-SSA antibodies, the risk factor for food allergy was ACD and vice versa, the risk factors for AR/AC were ACD, asthma, and high eosinophil levels, and the risk factor for asthma was AR/AC.

## Introduction

Sjögren's syndrome (SS) is a systemic autoimmune disease that affects the lacrimal and salivary glands and causes a chronic sicca syndrome.^1^ SS can be primary SS (pSS), which is not associated with other rheumatic diseases, or secondary SS, which is associated with other autoimmune diseases including rheumatoid arthritis (RA), systemic lupus erythematosus, or systemic sclerosis.^2^ Anti-Ro (anti–SS-related antigen A [SSA] autoantibodies) and/or anti-La antibodies (anti–SS–related antigen B autoantibodies) are almost always found in patients with pSS.^3^

A high prevalence of allergic disorders has been found in patients with SS.^4^ Nevertheless, the risk factors for allergic disorders in patients with pSS remain unclear. Takahashi et al^5^ reported that drug allergy may lead to asymptomatic SS and suggested that this may be a feature of SS.

This study aimed to compare the prevalence of allergic disorders in patients with pSS and RA. The secondary aim was to compare the clinical features of patients with pSS with and without drug allergy, food allergy, allergic contact dermatitis (ACD), allergic rhinitis (AR)/allergic conjunctivitis (AC), or asthma. Since we did not have data on the general population, patients with RA, who comprise a large population, were used as a control group. We also referred to previous reports on the prevalence of allergic disorders compared to RA in conditions such as SS,^4^ systemic lupus erythematosus,^6^ and adult-onset Still's disease,^7^ and compared them with those in RA in this study.

## Patients and methods

In this retrospective study, we analyzed data extracted from the medical records of consecutive patients diagnosed with pSS and RA at our hospital between 2010 and 2020. This study was approved by the institutional review board of our institution (approval number for National Tokyo Medical Center: R20-181), and the requirement of obtaining written informed consent from the patients was waived according to the regulations in Japan. All procedures were performed in accordance with the ethical standards of the institutional and national research committees and the 1975/1983 Helsinki Declaration and its later amendments. Patients with pSS met the 1999 revised Japanese Ministry of Health criteria^8^ and the 2016 American College of Rheumatology (ACR)/European League Against Rheumatism (EULAR) classification criteria.^9^ Patients with RA met the ACR/EULAR 2010 criteria.^10^ Patients with pSS who had Mikulicz disease, which has been associated with allergic disorders and shows a clinical pattern similar to SS, were excluded. We divided the participants into two groups: 1) the pSS group, which included patients diagnosed with pSS but not with RA or other rheumatic diseases; 2) the RA group, consisting of patients with RA who had no other rheumatic diseases.

To investigate the prevalence of allergic disorders in patients with pSS and RA, we performed our first analysis focused on 5 types of allergic reactions: (1) food allergy (rash, angioedema, and anaphylaxis after food exposure), (2) drug allergy (rash, angioedema, and anaphylaxis after drug exposure), (3) ACD caused by metals, synthetic fiber, rubber, and cosmetics, (4) AR and/or AC, and (5) asthma. We defined rash or angioedema associated with drug allergy as rash or angioedema that appears after drug ingestion and disappears after discontinuation of the drug. It was retrospectively distinguished from a symptom associated with pSS. We defined rash or angioedema associated with food allergy as rash or angioedema that appeared after food ingestion, was reproducible, and did not appear when the food was removed. It was also retrospectively distinguished from a symptom associated with pSS. ACD was retrospectively distinguished from a symptom associated with pSS by defining ACD as the appearance of symptoms at the skin of contact with metals, synthetic fibers, rubber, or cosmetics, which resolved after contact was discontinued. Angioedema induced by non-steroidal anti-inflammatory drugs was not considered an allergy because it is believed to be caused primarily by prostaglandin imbalance due to cyclooxygenase inhibition rather than by an allergic reaction. The prevalence of anxiety disorders in patients with pSS is higher than that in healthy individuals,^11^ and it may be difficult to determine the presence of allergy based only on patients' self-reported complaints. Therefore, this study included only medical record data from patients with allergic symptoms (rash, angioedema, anaphylaxis) confirmed by a physician after ingestion or contact with an allergen.

A second analysis was performed on patients' baseline laboratory data at the time of diagnosis of pSS to identify clinical patterns in patients with pSS in relationship to drug allergy, food allergy, ACD, AR/AC, and asthma. All data were analyzed by univariate and multivariate logistic regression analyses using JMP version 14.0 (SAS Institute, Cary, NC, USA). Univariate analysis, Fisher's exact test, and logistic regression analysis were used to evaluate the factors associated with allergic disorders. Results that did not follow the Gaussian distribution are expressed as the median of the 25–75th percentile (interquartile range); whereas results that followed the Gaussian distribution are expressed as mean ± standard deviation. The odds ratio (OR) and 95% confidence interval (CI) indicated an increased or decreased risk of drug allergy associated with a one-unit change in the predictor variable for continuous variables. For dichotomous variables, the OR indicated the risk of drug allergy associated with the presence of the feature compared with the absence of it. Receiver-Operator Characteristic (ROC) curves were generated for the significantly differentiated continuous variables, and the best values of cutoff were selected on the ROC curves.

## Results

A total of 292 and 413 Japanese patients with pSS and RA, respectively, were included in the first analysis (Table 1). The median age (in years) of patients with pSS at diagnosis was 58.0, and that of patients with RA was 68.0. Most of the patients (pSS, 94.2% and RA, 78.2%) were female. The median observation period was 68.0 and 64.0 months for patients with pSS and RA, respectively. None of the patients were using immunomodulators or immunosuppressive drugs at the time of diagnosis. As shown in Table 2, 54.8% of patients with pSS and 34.9% of patients with RA presented with at least one type of allergic disorder (*P* < 0.01). The allergic disorders included food allergy (pSS, RA: 12.0%, 6.8%; *P* = 0.02), drug allergy (26.4%, 16.2%; *P* < 0.01), ACD (3.4%, 4.6%; *P* = 0.6), AR/AC (33.9%, 8.5%; *P* < 0.01), and asthma (9.9%, 6.1%; *P* = 0.06). Food allergy, drug allergy, and AR/AC occurred more frequently in patients with pSS than in those with RA.

**Table 1:** Characteristics of patients with pSS and RA at diagnosis.

	pSS (n = 292)	RA (n = 413)	*P*
Female (n, %)	275 (94.2%)	323 (78.2%)	<0.01
Age (years)	58.0 (46.0–70.0)	68.0 (56.0–77.0)	<0.01
Length of follow-up (months)	68.0 (34.5–99.0)	64.0 (39.0–97.5)	0.33
History of immunosuppressant or steroid use (n, %)	0 (0%)	0 (0%)	–
White blood cell count (/μL)	4800 (3900–5900)	7000 (5800–8325)	<0.01
Eosinophil count (/μL)	114.6 (50.1–202.0)	118.8 (66.0–208.0)	0.15
Red blood cell count (10^4^/μL)	421.5 (391.0–450.8)	411.0 (378.0–441.3)	0.01
Hemoglobin level (g/dL)	12.8 (11.9–13.5)	12.5 (11.4–13.4)	0.01
Platelet count (10^4^/μL)	21.9 (18.9–25.9)	27.2 (22.0–33.3)	<0.01
Erythrocyte sedimentation rate (mm/h)	27.0 (16.5–45.5)	40.5 (23.0–68.0)	<0.01
Total protein level (g/dL)	7.5 (7.2–8.1)	7.2 (6.9–7.6)	<0.01
Albumin level (g/dL)	4.3 (4.1–4.4)	4.0 (3.7–4.3)	<0.01
Creatinine level (mg/dL)	0.64 (0.59–0.73)	0.65 (0.56–0.77)	0.64
Aspartate aminotransferase level (U/L)	22.0 (19.0–27.0)	19.0 (16.0–24.0)	<0.01
Alanine aminotransferase level (U/L)	17.0 (13.0–23.8)	15.0 (11.3–22.0)	<0.01
C-reactive protein level (mg/dL)	0 (0–0.1)	1.1 (0.3–3.0)	<0.01

Abbreviations: n, number of patients; pSS, primary Sjögren's syndrome; RA, rheumatoid arthritis

**Table 2:** Allergic disorders in patients with pSS and RA.

	pSS (n = 292)	RA (n = 413)	*P*
At least one type of allergy (n, %)	160 (54.8)	144 (34.9)	<0.01
Food allergy (n, %)	35 (12.0)	28 (6.8)	0.02
Drug allergy (n, %)	77 (26.4)	67 (16.2)	<0.01
ACD (n, %)	10 (3.4)	19 (4.6)	0.6
AR/AC (n, %)	99 (33.9)	35 (8.5)	<0.01
Asthma (n, %)	29 (9.9)	25 (6.1)	0.06

Abbreviations: ACD, allergic contact dermatitis; AR/AC, allergic rhinitis/allergic conjunctivitis; n, number of patients; pSS, primary Sjögren's syndrome; RA, rheumatoid arthritis

In the second analysis, we examined the characteristics of patients with pSS with (n = 77) and without (n = 215) drug allergies (Table 3). No significant difference in patients’ age at diagnosis of pSS was detected between both groups (median 56.5 and 58.0 years, for patients with and without drug allergy, respectively). Most of the patients in both groups were female (96.1% with drug allergy, 93.5% without drug allergy), and the median observation period was 76.0 and 63.0 months in patients with and without drug allergy, respectively. Patients with pSS who had drug allergy had significantly more food allergy (19.5%) and higher levels of eosinophils (159.5/μL) than those without drug allergy (9.3% and 97.4/μL), and a numerically higher positivity rate of anti-SSA antibody (89.6% vs. 79.7%; *P* = 0.056) than those without drug allergy. The cutoff value of eosinophils for drug allergy was 159.1/μL.

**Table 3:** Characteristics of patients with pSS with and without drug allergy.

	With drug allergy (n = 77)	Without drug allergy (n = 215)	*P*
Female (n, %)	74 (96.1%)	201 (93.5%)	0.57
Age (years)	56.5 (44.5–70.0)	58.0 (46.5–70.0)	0.45
Length of follow-up (months)	76.0 (41.0–110.0)	63.0 (31.0–96.5)	0.09
History of antihistamine use (n, %)	9 (11.7%)	22 (10.2%)	0.67
At least one type of allergic disorder other than drug allergy (n, %)	46 (59.7%)	82 (38.1%)	<0.01
Food allergy (n, %)	15 (19.5%)	20 (9.3%)	0.02
ACD (n, %)	4 (5.2%)	6 (2.8%)	0.30
AR/AC (n, %)	33 (42.9%)	66 (30.7%)	0.07
Asthma (n, %)	8 (10.4%)	21 (9.8%)	0.83
White blood cell count (/μL)	4950.0 (3975.0–6500.0)	4800.0 (3850.0–5900.0)	0.54
Eosinophil count (/μL)	159.5 (75.0–280.0)	97.4 (40.7–188.8)	<0.01
Red blood cell count (10^4^/μL)	418.0 (391.0–443.0)	422.0 (391.0–454.0)	0.51
Hemoglobin level (g/dL)	12.4 (12.0–13.3)	12.8 (11.9–13.6)	0.32
Immunoglobulin level^a^ (mg/dL)	1733.5 (1348.0–2170.8)	1560.0 (1331.0–2036.8)	0.13
C-reactive protein level (mg/dL)	0.1 (0–0.1)	0 (0–0.1)	0.18
Patients with anti-SSA antibody (n, %)	69 (89.6%)	169 (79.7%)	0.056
Patients with antinuclear antibody^b^ (n, %)	63 (82.9%)	177 (84.3%)	0.86

Abbreviations: ACD, allergic contact dermatitis; anti-SSA antibody, anti-Sjögren's-syndrome-related antigen A autoantibody; AR/AC, allergic rhinitis/allergic conjunctivitis; CI, confidence interval; n, number of patients; OR, odds ratio; pSS, primary Sjögren's syndrome.

a 72 and 202 and.

b 76 and 210 patients with and without drug allergy, respectively

The most common triggers of drug allergy were antimicrobial drugs (Table 4). Antimicrobials, including penicillin, were common triggers for patients with pSS, and immunomodulators, including salazosulfapyridine, and antimicrobials were common triggers for patients with RA.

**Table 4:** Triggers of drug allergy.

Drug	pSS (n = 292)	RA (n = 413)
*Antimicrobial drugs*	37	15
Penicillin antibiotics	10	7
Cephem antibiotics	9	5
Tetracycline antibiotics	2	3
Macrolide antibiotics	6	0
New quinolone antibiotics	3	1
Glycopeptide antibiotics	1	0
Sulfamethoxazole/trimethoprim	2	1
Vonoprazan, amoxicillin, and clarithromycin	1	0
Anti-tuberculosis drugs	1	1
Anti-herpetic drugs	0	1
Anti-influenza drugs	2	0
Unknown	1	0
*Immunomodulators*	3	21
Salazosulfapyridine	3	15
Busiramine	0	5
Iguratimod	0	1
*Immunosuppressants*	3	1
Mizoribine	3	0
Methotrexate	0	1
Azathioprine	0	0
*Biological drugs*	0	6
Etanercept	0	1
Abatacept	0	1
Tocilizumab	0	3
Certolizumab pegol	0	1
*Vaccine*	3	0
*Others*	45	28

Abbreviations: n, number of patients; pSS, primary Sjögren's syndrome; RA, rheumatoid arthritis

**Fig. 1:**
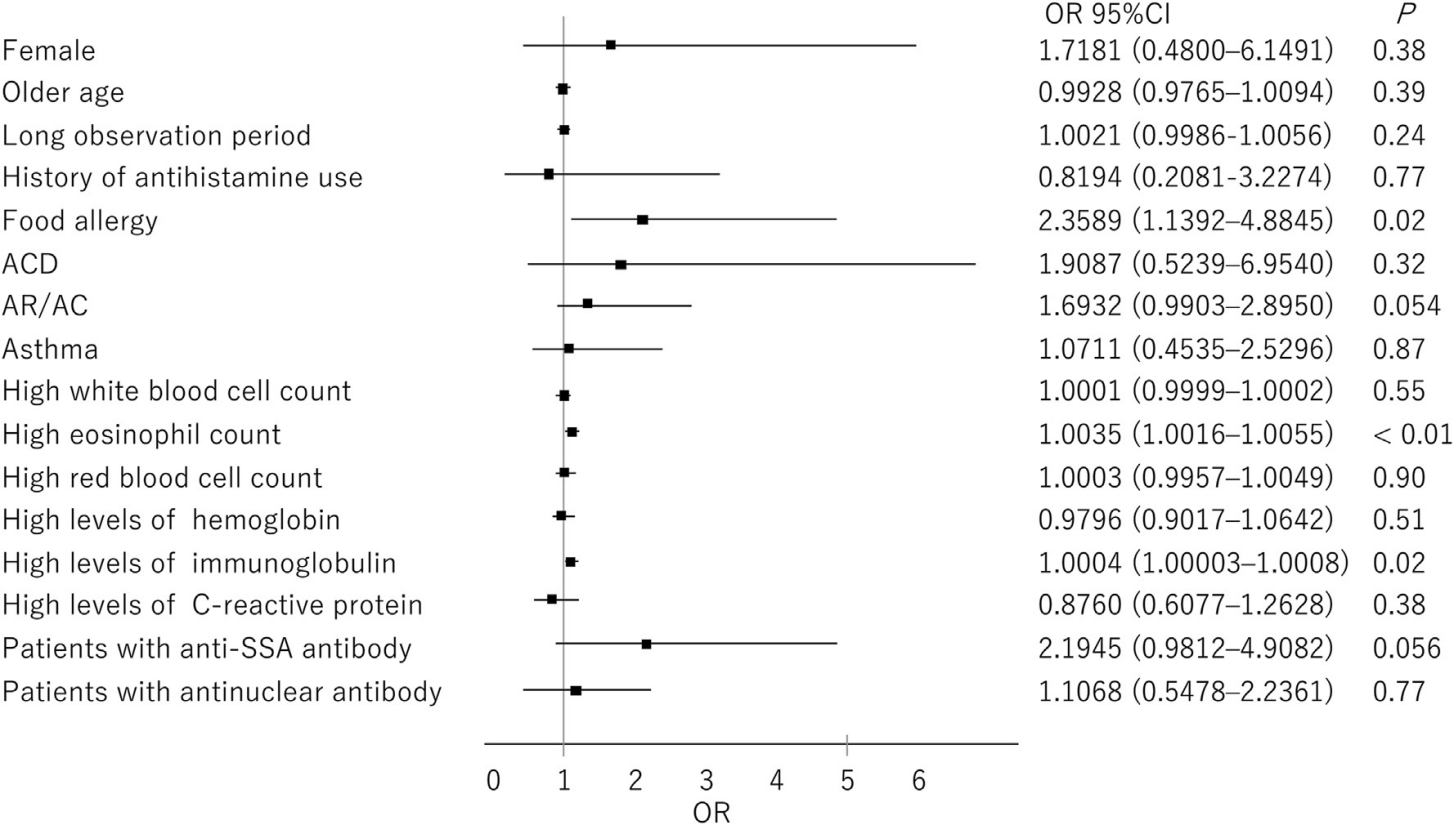
Risk factors for drug allergy in patients with pSS analyzed using univariate logistic regression analysis. Abbreviations: ACD, allergic contact dermatitis; anti-SSA antibody, anti–SS-related antigen A antibody; AR/AC, allergic rhinitis/allergic conjunctivitis; CI, confidence interval; OR, odds ratio; pSS, primary Sjögren's syndrome

**Fig. 2:**
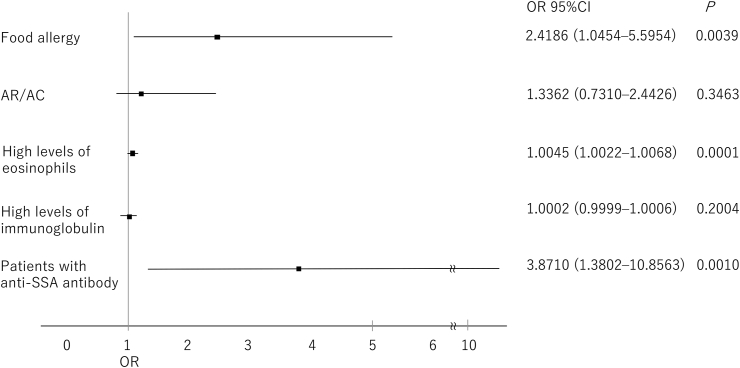
Risk factors for drug allergy in patients with pSS analyzed using multivariate logistic regression analysis. Abbreviations: anti-SSA antibody, anti–SS-related antigen A antibody; AR/AC, allergic rhinitis/allergic conjunctivitis; CI, confidence interval; OR, odds ratio; pSS, primary Sjögren's syndrome

In the pSS group, univariate analyses showed that food allergy (*P* = 0.02), AR/AC (*P* = 0.054), high levels of eosinophils (*P* < 0.01), high levels of immunoglobulins (*P* = 0.02), and positivity for anti-SSA antibody (*P* = 0.056) were risk factors for drug allergy (Fig. 1). In the same group, multivariate analyses showed that food allergy (*P* < 0.01), high levels of eosinophils (*P* < 0.01) and anti-SSA antibody positivity (*P* < 0.01) were risk factors for drug allergy (Fig. 2).

The characteristics of patients with allergic disorders other than drug allergy in the multivariate analysis are shown in Supplemental Figures 1, 2, 3, and 4.

In the univariate analysis, patients with pSS who had food allergy had significantly higher rates of drug allergy, ACD, and AR/AC and higher levels of eosinophils than those without food allergy. The multivariate analysis showed that ACD (OR 4.9282, 95% CI 1.1773–20.6287; *P* = 0.03) was significantly associated with food allergy (Supplemental Figure 1).

In the univariate analysis, patients with pSS who had ACD showed a significantly higher prevalence of food allergy and AR/AC than those without ACD. The multivariate analysis showed that food allergy (OR 4.2521, 95% CI 1.1016–16.4127; *P* = 0.04) was significantly associated with ACD (Supplemental Figure 2).

In the univariate analysis, patients with pSS who had AR/AC had a significantly higher prevalence of food allergy, ACD, and asthma and higher levels of eosinophils than those without AR/AC. The multivariate analysis showed that ACD (OR 6.0975, 95% CI 1.1828–31.4330; *P* = 0.03), asthma (OR 2.6765, 95% CI 1.1855–6.0429; *P* = 0.02), and high eosinophil levels (OR 1.0017, 95% CI 1.00002–1.0034, *P* = 0.048) were significantly associated with AR/AC (Supplemental Figure 3). The cutoff value of eosinophils for AR/AC was 257.4/μL.

In the univariate analysis, patients with pSS who had asthma had a significantly higher prevalence of AR/AC and higher eosinophil levels than those without asthma. The multivariate analysis showed that AR/AC (OR 2.8117, 95% CI 1.2639–6.2552, *P* = 0.01) was significantly associated with asthma (Supplemental Figure 4).

These risk factors for drug allergy, food allergy, ACD, AR/AC, and asthma in patients with pSS were not risk factors in patients with RA.

## Discussion

The prevalence of food allergy, drug allergy, AR, AC, and asthma in the general population has been reported to be approximately 3–4%,^12^ 3.6–7%,^13^ 9%,^14^ 14.8%,^15^ and 4.2%,^16^ respectively. Our study indicates that the prevalence of the abovementioned allergic disorders in patients with pSS and the prevalence of food allergy, drug allergy, and asthma in patients with RA may be higher than those in the general population, although the prevalence of these disorders in the general population was not directly compared with that in patients with pSS/RA in this study.

Previous reports have shown that the prevalence of allergic disorders in patients with SS was 51%,^4^ 43%,^17^ 8%,^4^ 33.0%,^4^ and 14.0%,^4^ for drug allergy, food allergy, ACD, and AR/asthma, respectively. For patients with SS, the prevalence of drug allergy and ACD was slightly lower in our study than previously reported. We believe that the differences could be attributed, at least in part, to differences in racial background, frequency of drug exposure, and environmental factors.

Furthermore, reports have shown that the prevalence of allergic disorders in patients with RA was 15%,^4^ 6%,^4^ 24.0%,^18^ and 31.2%^18^ for drug allergy, food allergy, bronchial asthma, and AR, respectively. In our study, bronchial asthma and AR/AC were slightly less common among patients with RA than previously reported, which we believe could also be attributed to differences in racial background and environmental factors.

In our study, patients with pSS presented a higher prevalence of food allergy, drug allergy, and AR/AC than patients with RA. These results are consistent with those of Tishler et al.,^4^ who indicated that pSS was associated with a significantly higher prevalence of drug allergy and skin contact allergy than RA.

Kim et al^19^ reported that dry eyes were associated with AC and sensitization to allergens. Patients with SS who had dry eyes have more opportunities to be sensitized than patients with RA, which may be linked to the higher prevalence of AC in them.

Drug allergy in patients with SS has been reported to be associated with human leukocyte antigen (HLA)-DR3 positivity.^17^ Furthermore, the number of Tregs in the peripheral blood mononuclear cells of patients with SS is significantly lower than that in the general population.^20^ Moreover, previous studies indicated that the regulatory function of Tregs may be impaired in patients with SS.^21^ When drugs are administered to patients with impaired Treg function, the T cells that react to the drugs are more likely to be activated and drug allergy are more likely to occur than in those without impaired Treg function.^22^ In our study, the most common triggers of drug allergy were antimicrobial drugs in patients with pSS. A common drug causing drug allergy is antibiotics in the general population,^23^ and Treg dysfunction and drug allergy are likely to occur in the presence of infection,^22^ even if not limited to patients with pSS. Since infections are more likely to cause Treg dysfunction and drug allergy, patients with pSS, who had Treg dysfunction and decreased numbers of Tregs, may be more likely to be allergic to antimicrobial agents at the time of infection.

Since an association between food allergy and drug allergy has been reported,^24^ patients with pSS who had food allergy may have a higher prevalence of drug allergy than those without food allergy.

The univariate analysis revealed that patients with pSS who had drug allergy had a higher prevalence of food allergy and AR/AC, higher levels of eosinophils and immunoglobulin, and more patients with anti-SSA antibody positivity than those without drug allergy. In the multivariate analysis, food allergy, high levels of eosinophils and anti-SSA antibody positivity were associated with drug allergy.

These results are in accordance with those of Chambel et al^24^ and Martins et al^25^ Although the previous reports did not specifically indicate the triggers of food allergy, in our study, fruits, crustaceans, and fish were common triggers (Table 5).

**Table 5:** Triggers of food allergy.

Food	pSS (n = 292)	RA (n = 413)
Fruits	14	6
Shellfish	9	9
Fish	7	9
Egg	3	3
Wheat	3	1
Dairy products	2	2
Tree nuts	2	0
Bamboo shoot	2	0
Peanuts	1	1
Buck wheat	1	1
Vegetable	1	0
Mugwort	1	0
Chicken	1	0
Coffee	1	0
Spice	1	0
Yam	0	1

Abbreviations: n, number of patients; pSS, primary Sjögren's syndrome; RA, rheumatoid arthritis

Tishler et al^4^ reported that patients with pSS who have allergic disorders are more likely to be positive for anti-SSA antibodies than those without allergic disorders. Anti-SSA antibodies were first detected in the sera of patients with SS in 1975.^26^ The role of these antibodies in the development of SS has been addressed.^27^, ^28^, ^29^ The antigen recognized by the anti-SSA antibody is a complex comprising Ro60 and Ro52.^27^ Ro60 is exposed on the surface of apoptotic cells.^28^ RNA bound to this subunit is recognized by toll-like receptor (TLR)7/TLR8, and this receptor activation leads to the production of type I interferon, which initiates inflammation and is thought to be associated with the development of SS.^28^ Nagase et al.^29^ reported that TLR-mediated activation of inflammatory cells may lead to the exacerbation of allergic disorders. In our study, anti-SSA antibody positivity was a risk factor for drug allergy. In a study by Tishler et al.,^4^ 80% of patients with pSS who had allergic disorders also had drug allergy. Taken together, these results suggest an association between drug allergy and anti-SSA antibodies, and further studies would be needed to elucidate this relationship.

European-American patients with systemic lupus erythematosus who are allergic to sulfa drugs such as trimethoprim-sulfamethoxazole are more likely to have anti-Ro (SSA) antibodies.^30^ In our study, drug allergy to trimethoprim-sulfamethoxazole was observed in 10% of the patients with pSS who used trimethoprim-sulfamethoxazole. It has also been reported that patients with RA who experience adverse effects from d-penicillamine are more prone to have anti-Ro (SSA)-positive RA than anti-Ro (SSA)-negative patients.^31^^,^^32^ In our study, no patients with pSS or RA had a history of d-penicillamine use.

The observation that ACD had an effect on food allergy in patients with pSS supports previous studies that showed a relationship between allergic dermatitis and food allergy.^33^ ACD is mainly type IV allergy, but immunological contact urticaria and protein contact dermatitis are partially related to antigen-specific IgE and type I allergy.^34^^,^^35^ Schichter-Konfino et al^36^ reported milk protein-induced ACD in patients with IgE-mediated milk allergy. Lack et al^33^ reported that rashes following skincare with peanut oil possibly occurred due to percutaneous sensitization to the allergen and were associated with the development of peanut allergy.

The multivariate analysis indicated that food allergy influenced ACD in patients with pSS. Atopic dermatitis is a risk factor for the development of food allergy, and sensitization to allergens may occur through the application of allergens to inflamed skin.^33^

Our study shows that ACD, asthma, and high levels of eosinophils associated with AR/AC in patients with pSS. There was a report that 67% of patients with ICU or PCD types of ACD had AR.^37^ Patients with AR are known to have high levels of eosinophils^38^ and asthma.^39^

Moreover, we showed that AR/AC associated with asthma in patients with pSS. This supports the results of a previous study,^39^ which reported that 60–78% of patients with asthma had AR and that asthma and AR had inflammation involving the same airways.

Our study has some limitations: 1) it was difficult to determine the potential incidence of drug allergy because the drugs used differed in each patient; 2) the drug triggering the allergy was not confirmed (ie, there was no second exposure of the patient to the drug); 3) drug allergy was diagnosed based on information provided by the patient and information in the medical records; 4) it was difficult to categorize drug allergies into type I and type IV hypersensitivity reactions due to the retrospective study design and because data were mainly collected from the patients’ medical records; 5) allergen-specific IgE levels were not examined in this study but should be investigated in the future. However, these limitations equally affected the patients with pSS and those with RA, and we, therefore, believe that the sources of bias had a minimal effect on the results obtained.

Unless they presented with complications, patients with pSS were not treated with medications, whereas patients with RA were usually started on drugs as soon as they were diagnosed. Therefore, patients with RA, who were administered more drugs or for longer periods of time than patients with pSS, would have higher frequencies of drug allergy. However, in our study, the prevalence of drug allergy was higher in patients with pSS than in patients with RA. HLA-DR3 positivity was not assessed in our study. In the future, we will examine the relationship between HLA-DR3 positivity and drug allergy.

## Conclusions

Patients with pSS had a higher prevalence of allergic disorders than those with RA. Moreover, patients with pSS who had drug allergy had higher rates of food allergy, higher levels of eosinophils and higher positivity rates of anti-SSA antibodies than those without drug allergy. Interestingly, patients with pSS who had food allergy had a higher rate of ACD than those without food allergy, those with ACD had a higher rate of food allergy than those without ACD, those with AR/AC had a higher rate of ACD and asthma and higher levels of eosinophils than those without AR/AC, and those with asthma had a higher rate of AR/AC and higher levels of eosinophils than those without asthma. Further studies to investigate these associations are needed.

## Abbreviations

AC, allergic conjunctivitis; ACD, allergic contact dermatitis; ACR, American College of Rheumatology; AR, allergic rhinitis; CI, confidence interval; EULAR, European League Against Rheumatism; HLA, human leukocyte antigen; OR, odds ratio; pSS, primary Sjögren's syndrome; RA, rheumatoid arthritis; SS, Sjögren's syndrome; SSA, SS-related antigen A; TLR, toll-like receptor

## Financial support

The study has not been funded.

## Declaration of competing interest

The authors declare that they have no conflict of interest.

## Agreement to publish the work

We agree to publish the work.

## Author contributions

Conceptualization, M.H.-K. and K.I.; methodology, M.H.-K. and K.I.; software, M.H.-K.; validation, M.H.-K., K.I., T.S., S.H., T.O., and H.O.; formal analysis, M.H.-K.; investigation, M.H.-K.; data curation, M.H.-K.; writing—original draft preparation, M.H.-K.; writing—review and editing, M.H.-K. and K.I.; visualization, M.H.-K.; supervision, Y.O.; project administration, M.H.-K. All authors have read and agreed to the published version of the manuscript.

## Data availability statement

Not available.

## Ethics statement

This study was approved by the institutional review board of the National Hospital Organization Tokyo Medical Center (approval number: R20-181), and the requirement of obtaining written informed consent from the patients was waived according to the regulations in Japan.

## Editorial policy confirmation and agreement

We confirm editorial policy and agree.
