# Supplementary material for: Allergic disorders and their risk factors in primary Sjögren's syndrome
Source: World Allergy Organ J. 2023 Jan 27;16(2):100745. doi: 10.1016/j.waojou.2023.100745 (PMC9922985; doi:10.1016/j.waojou.2023.100745)
Supplement: Multimedia component 1 [file mmc1.pdf]

Supplemental Figure 1. Risk factors for food allergy in patients with pSS analyzed using multivariate logistic regression analysis

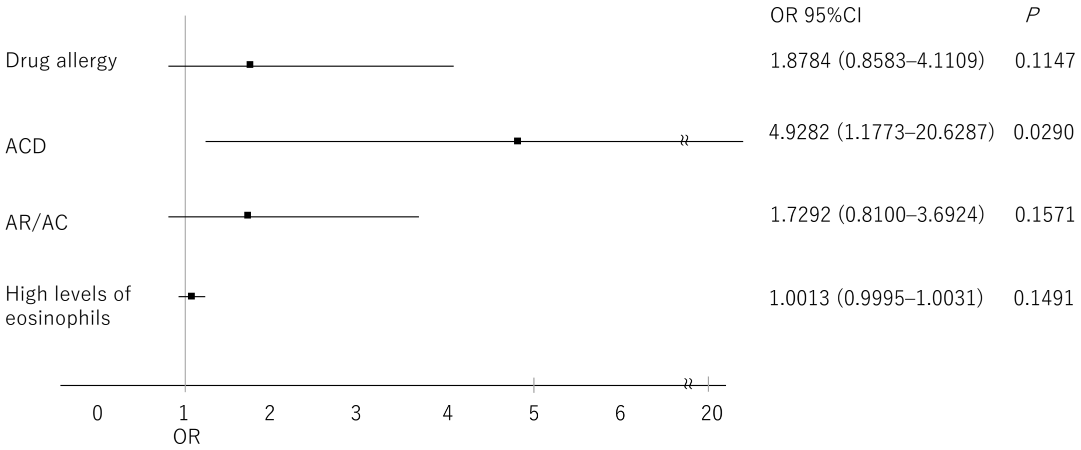

Abbreviations: ACD, allergic contact dermatitis; AR/AC, allergic rhinitis/allergic conjunctivitis; CI, confidence interval; OR, odds ratio; pSS, primary Sjögren's syndrome.

Supplemental Figure 2. Risk factors for ACD in patients with pSS analyzed using multivariate logistic regression analysis

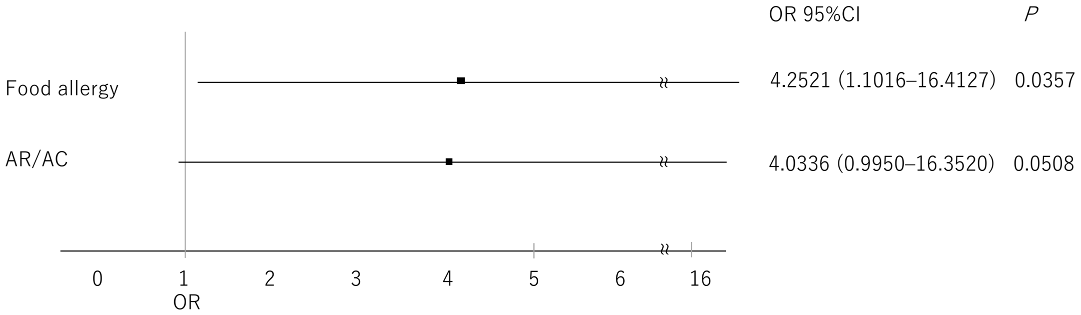

Abbreviations: ACD, allergic contact dermatitis; AR/AC, allergic rhinitis/allergic conjunctivitis; CI, confidence interval; OR, odds ratio; pSS, primary Sjögren’s syndrome.

Supplemental Figure 3. Risk factors for AR/AC in patients with pSS analyzed using multivariate logistic regression analysis

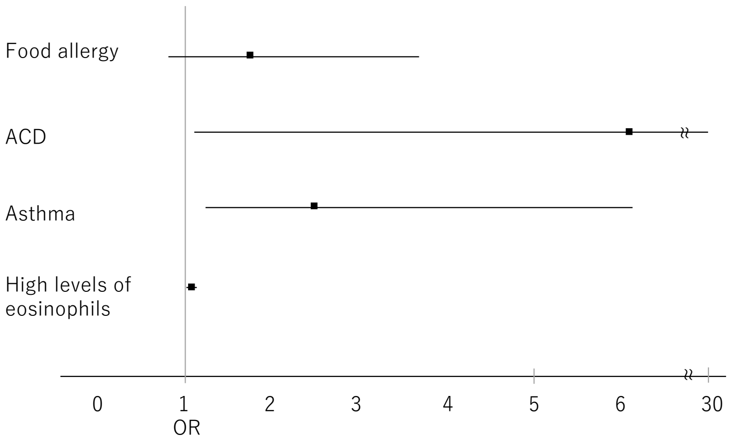

	OR 95%CI	*P*
Food allergy	1.8260 (0.8502–3.9223)	0.1226
ACD	6.0975 (1.1828–31.4330)	0.0307
Asthma	2.6765 (1.1855–6.0429)	0.0178
High levels of eosinophils	1.0017 (1.00002–1.0034)	0.0477

Abbreviations: ACD, allergic contact dermatitis; AR/AC, allergic rhinitis/allergic conjunctivitis; CI, confidence interval; OR, odds ratio; pSS, primary Sjögren’s syndrome.

Supplemental Figure 4. Risk factors for asthma in patients with pSS analyzed using multivariate logistic regression analysis

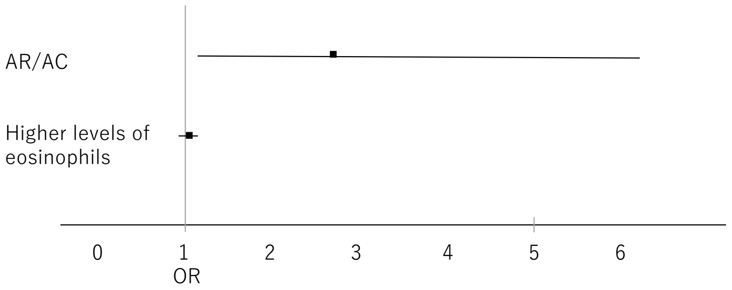

	OR 95%CI	*P*
AR/AC	2.8117 (1.2639–6.2552)	0.0113
Higher levels of eosinophils	1.0015 (0.9997–1.0033)	0.0967

Abbreviations: AR/AC, allergic rhinitis/allergic conjunctivitis; CI, confidence interval; OR, odds ratio; pSS, primary Sjögren's syndrome.
